# Longitudinal Links Between Media Use and Focused Attention Through Toddlerhood: A Cumulative Risk Approach

**DOI:** 10.3389/fpsyg.2020.569222

**Published:** 2020-11-02

**Authors:** Noa Gueron-Sela, Avigail Gordon-Hacker

**Affiliations:** ^1^Department of Psychology, Ben-Gurion University of the Negev, Beer-Sheva, Israel; ^2^Zlotowski Center for Neuroscience, Ben-Gurion University of the Negev, Beer-Sheva, Israel

**Keywords:** media use, early childhood, focused attention, cumulative risk, background television, screen time, parental media use

## Abstract

Previous studies that examined the links between media use and children’s attention abilities have yielded inconclusive findings. In the current study, we aimed to move beyond the focus on isolated aspects of media use to a comprehensive assessment of both direct and indirect media use and practices in early childhood. Drawing from the cumulative risk literature, we examined whether cumulative media use is related to children’s subsequent attention abilities. Participants were 199 mothers of toddlers (60% male) who completed questionnaires assessing various aspects of children’s media use, as well as children’s focused attention abilities at three time points: 18 months (T1), 22 months (T2), and 26 months (T3) of age. Cumulative media use scores were computed based on four indicators: (1) child average daily screen time; (2) household background television; (3) maternal use of media to regulate child distress; and (4) maternal use of mobile devices while spending time with the child. An autoregressive cross-lagged (ARCL) path model controlling for child sex, maternal education, and general parenting practices showed that cumulative media use at 18 months negatively predicted children’s focused attention at 22 months. Moreover, there was a significant negative indirect effect from cumulative media use at 18 months to focused attention at 26 months via focused attention at 22 months. Finally, the cumulative media index appeared to be a better predictor of focused attention than any of the singular media use indicators. Children’s focused attention did not predict subsequent cumulative media use across time, providing no evidence for bidirectional links. Findings suggest that exposure to multiple (rather than single) aspects of media use is related to decreased subsequent focused attention abilities during toddlerhood. Family media plans that designate media-free time and increase parental awareness to media use habits in the household should therefore be encouraged.

The relationship between children’s use of screen-based media and attention abilities has been a primary focus of research for over four decades (Nikkelen et al., 2014; Kostyrka-Allchorne et al., 2017). During this period, children’s media content has become more fast-paced, arousing, and easily accessible to very young children, leading to the development of several hypotheses regarding how these aspects of media use could hamper children’s developing attentional skills (Nikkelen et al., 2014). However, despite the accumulation of research on this topic, the extent to which screen media use and attention abilities are linked remains unclear due to a considerable amount of mixed findings (Landhuis et al., 2007; Foster and Watkins, 2010). Notably, the vast majority of these studies have focused on isolated aspects of media use, mainly the *amount* of exposure to screen media, overlooking the importance of contextual factors of media use (Barr, 2019). Family media ecology refers to the way media are used by all members of the household, including children’s direct and indirect exposure, and how media are used in children’s daily routines such as play, discipline, meals, and bedtime (Barr, 2019). Guided by this contextual framework, in the current study we applied a comprehensive assessment of media use and practices in early childhood. Drawing from the cumulative risk literature suggesting that multiple risk factor exposure exceeds the adverse developmental impacts of singular exposures (Evans et al., 2013), we examined whether cumulative media use is related to children’s emerging focused attention abilities. We specifically focused on four indicators of media use and exposure that were selected based on recommendations of the American Academy of Pediatrics (AAP) for media use in early childhood (Council on Communications, and Media, 2016) and previous research linking these indicators to attention abilities directly or indirectly (Kirkorian et al., 2009; Radesky et al., 2016; Kildare and Middlemiss, 2017; Kostyrka-Allchorne et al., 2017), including daily screen time, household background television, use of media to regulate child distress, and parental use of mobile devices while spending time with the child.

## Focused Attention Across Toddlerhood

Focused attention, defined as the ability to sustain attention during active engagement with a stimulus or task, is one of the primary attentional skills that enable response persistence, cognitive information processing, and goal-directed behavior (Ruff and Rothbart, 1996; Garon et al., 2008). A substantial body of literature has addressed the role of focused attention in learning and cognitive development, finding that focused attention abilities during infancy and toddlerhood are predictive of later general cognitive abilities and executive function (Lawson and Ruff, 2004; Johansson et al., 2015). The development of sustained attention during early childhood is attributed in part to the development of two attention subsystems: the orienting system, which allows children to attend to stimuli in the external environment, and the executive attention network, which enables more volitional control of attention and the ability to focus attention in the face of potential distractions (Ruff and Rothbart, 1996; Posner et al., 2014). The gradually increasing dominance of the executive attention network toward the end of the first year of life supports children’s emerging ability to sustain attention for prolonged periods of time (Colombo and Cheatham, 2007). Indeed, research has shown significant increases in children’s duration and frequency of sustained attention during free play and structured situations from late infancy to early childhood (Ruff and Lawson, 1990; Ruff and Capozzoli, 2003; Kannass et al., 2006).

The development of attention abilities has strong biological underpinnings that are considered constitutional and genetic in origin but is also shaped by children’s environmental experiences (Colombo and Salley, 2015). The increase in young children’s screen media exposure over the past two decades has led to concern about the impact of screen media exposure on the development of the attention networks (Nikkelen et al., 2014; Courage, 2017). Consequently, a substantial body of literature has focused on the links between screen media use and two main aspects of attention measured in the preschool period: attention problems (e.g., distractibility, inability to focus) and executive function (EF; i.e., inhibitory control, working memory, and cognitive set shifting). However, little is known about the potential impact of screen media in toddlerhood, a period when children may be particularly susceptible to environmental experiences that support or hinder the development of attention networks (Comas et al., 2014; Gueron-Sela et al., 2018). Due to the recent increases in screen media exposure in this developmental time period (Rideout, 2017), the current study focused on three time points across toddlerhood: 18, 22, and 26 months. We chose focused attention as our main outcome because it is considered the foundation for the development of EF abilities in later childhood (Garon et al., 2008) and is also predictive of later attention problems (Miller et al., 2018).

## Cumulative Media Exposure and Focused Attention

The concept of cumulative risk has gained considerable attention within developmental science, mainly due to the robust finding that children who experience multiple and cumulative risk factors in early life show more adverse developmental outcomes than those who experience singular risk factors (Evans et al., 2013; Gach et al., 2018). Traditionally, cumulative risk approaches have been used to assess socioeconomic risk, including financial factors (e.g., low income), family resources (e.g., poor total family functioning), and parental personal resources (e.g., poor mental health; Evans et al., 2013). In the current study, we aimed to apply a similar approach to assess media use in early childhood while considering multiple aspects of exposure to media. In the following paragraph, we describe the individual factors that comprised the cumulative media use (CMU) index and explain how they are related to children’s attention abilities. Our choice of indicators was informed by recommendations provided by the AAP regarding media use in early childhood (Council on Communications, and Media, 2016) and by previous literature linking screen media exposure and attention abilities either directly (Kostyrka-Allchorne et al., 2017) or indirectly through parent–child interactions (Kirkorian et al., 2009) and children’s self-regulation abilities (e.g., Radesky et al., 2016).

### Screen Time

Based on studies showing associations between excessive television viewing in early childhood and cognitive, language, and social–emotional delays, the 2016 AAP guidelines recommend that screen media exposure be limited to no more than 1 h per day for 2–5-year-old children to allow sufficient time to engage in other activities important to their development (Council on Communications, and Media, 2016). However, nationally representative data from the United States indicate that 2–4-year-old children are exposed to more than 2 h per day on average (Rideout, 2017). These numbers have raised concerns regarding the effects of excessive exposure to screen media on children’s cognitive development, and particularly their attention abilities (Anderson and Subrahmanyam, 2017; Courage, 2017). Indeed, the vast majority of empirical studies that examined the associations between media use and attention have used the total amount of direct exposure to screen media as an indicator of children’s media use and focused mainly on attention problems as an outcome (see Kostyrka-Allchorne et al., 2017 for review). Overall, whereas there is some evidence for positive cross-sectional links between screen time and attention problems in early childhood (Tamana et al., 2019), longitudinal studies that considered the bidirectional links between screen time and attention problems over time have generally found no support for such links (Stevens et al., 2009; Foster and Watkins, 2010).

Other studies have focused on the links between screen media use and children’s EF. Findings from these studies suggest overall that higher screen time may be related to poorer EF abilities in the preschool period (Barr et al., 2010; Nathanson et al., 2014). However, the nature of this association is complex and depends on factors such as children’s age, parenting practices, type of programming watched, and demographic factors (Barr et al., 2010). For example, Barr et al. (2010) found that only high levels of exposure to adult-directed (but not child-directed) media content were associated with poorer EF at age 4. Linebarger et al. (2014) further demonstrated that for children at high demographic risk, increased exposure to educational media content was associated with better EF. Finally, in one study the amount of television viewing was negatively related to EF at age 5, but this association was no longer significant when controlling for the home learning environment and parental scaffolding (Blankson et al., 2015).

### Household Background Television

The AAP advises parents to reduce young children’s exposure to background television (i.e., adult-directed content to which children pay little active attention; Anderson and Evans, 2001) in the household, as it can be distracting and interfere in experiences such as toy play and social interactions that are essential for children’s cognitive development (Anderson and Pempek, 2005; Council on Communications, and Media, 2016). Indeed, experimental research directly assessing the impact of background television indicates that it disrupts children’s attention during play (Schmidt et al., 2008; Setliff and Courage, 2011). For example, in the presence of background television, young children (ages 12, 24, and 36 months) showed less solitary toy play overall and shorter bouts of focused attention than in play situations in which the television was off (Schmidt et al., 2008).

An additional body of research focused on correlational links between background television and children’s EF (Barr et al., 2010; Linebarger et al., 2014). For example, Barr et al. (2010) showed that children’s high levels of exposure to household television during infancy and at age four were associated with poorer EF at age four (Barr et al., 2010). Similarly, Linebarger et al. (2014) showed that greater exposure to background television was associated with lower EF for preschool children at high demographic risk, and low-risk primary school children. Parenting style further moderated the latter relationship, with high levels of inconsistent parenting behaviors exacerbating the negative effects of background television on EF. Findings from this study showed that the associations between background television and EF are complex and may depend on additional factors such as demographic risk and parenting (Linebarger et al., 2014).

The impact of background television on parental behavior can also be a mechanism through which background television can impede children’s attention skills. During infancy and toddlerhood, dyadic social interactions serve as a primary socialization mechanism in which parents engage to support their infants’ attention abilities (Yu and Smith, 2016). Through time, continuous shared attentional states between the parent and the child can facilitate children’s ability to sustain attention toward objects on their own for increasingly longer stretches of time (Yu and Smith, 2016). The distractions caused by the presence of background television can disrupt this process. For example, in the presence of background television, parents were found to be less verbally interactive with their children and less responsive to their children’s bids for attention than when the television was off (Kirkorian et al., 2009).

### Use of Media to Regulate Child Distress

Parents often report using screens to soothe their children (Kabali et al., 2015; Radesky et al., 2016; Gordon-Hacker and Gueron-Sela, 2020). However, the AAP recommends not relying heavily on screen media devices to regulate children’s distress, as excessive use of this strategy could interfere with the development of children’s self-regulation abilities (Council on Communications, and Media, 2016). During early childhood, self-regulatory abilities are limited and children largely depend on external regulation provided by their parents in modulating their arousal (Sameroff, 2010). When parents respond to children’s negative emotions in unsupportive ways, such as punitive reactions, personal distress, or minimizing the child’s distress, children may experience hyperarousal, which can interfere with their ability to focus and shift attention in response to environmental demands (Spinrad et al., 2007). Indeed, unsupportive maternal responses to children’s negative emotions were negatively related to children’s later attentional control (Spinrad et al., 2007). The use of media to soothe negative emotions may establish passive and ineffective regulatory strategies in young children, resulting in increased arousal and difficulties in regulating and focusing attention for prolonged periods of time.

### Parental Mobile Device Use

Finally, based on research showing that heavy parental use of mobile devices is associated with fewer verbal and non-verbal interactions between parents and children (e.g., Radesky et al., 2015), which are essential for children’s cognitive and social–emotional development, the AAP recommends reducing parental media use while parenting and enhancing parent–child “media free” interactions (Council on Communications, and Media, 2016). Accumulating evidence suggests that when parents are occupied with mobile devices, their ability to respond to their children’s cues is limited (see Kildare and Middlemiss, 2017 for a review). Similar to background television, parental use of mobile devices may interfere with parent–child reciprocal social interactions that serve as a primary socialization mechanism for the development of attention skills. For example, research has found that mothers distracted by mobile devices exhibited less verbal and non-verbal communication with their children, were slower to respond to their children’s engagement attempts, and were less sensitive in their eventual responses than were mothers who were not engaged with a device (Radesky et al., 2014a; Hiniker et al., 2015). On the child’s side, children showed less toy engagement when their mothers were occupied with a mobile device than during free play with no mobile device (Myruski et al., 2018). Thus, excessive parental mobile device can result in continuous disruptions in parent–child social interactions that prevent children from practicing their emerging focused attention skills.

## The Current Study

Given the increase in screen media use in the past decade by both parents and young children, understanding the potentially harmful implications for children’s cognitive abilities is critical (Anderson and Subrahmanyam, 2017; Courage, 2017). The current study addressed this issue by examining the links between a cumulative index of media use and children’s focused attention abilities at three time points in toddlerhood: 18 (T1), 22 (T2), and 26 (T3) months of children’s age. We aimed to expand extant literature in three main ways. First, guided by a family media ecology framework and the recent call to broaden the examination of media effects beyond screen time (Barr, 2019), we examined four different aspects of media use in early childhood that can be related to children’s attention abilities, including overall screen time (Nikkelen et al., 2014), background television (Anderson and Pempek, 2005; Schmidt et al., 2008; Courage, 2017), use of media to regulate child distress (Radesky et al., 2016), and mobile device use while parenting (Kildare and Middlemiss, 2017). Second, we applied a cumulative risk approach that can be especially helpful in assessing the additive impact of multiple sources of exposure that span a variety of children’s daily experiences. Finally, acknowledging the potential bidirectional links between media use and child characteristics (Radesky et al., 2014b; Kostyrka-Allchorne et al., 2017; Cliff et al., 2018), we used a short-term longitudinal design that enabled us to disentangle the transactional links between media use and attention abilities. Importantly, because these indicators of media use may tap into general parenting practices, we controlled for maternal supportive and unsupportive parenting behaviors in order to elucidate the unique implications of media use for children’s attention abilities.

We hypothesized that CMU and child-focused attention would show both prospective and longitudinal negative associations between T1, T2, and T3. We also examined whether the CMU index is a more powerful predictor of focused attention than any of the singular factors that comprise the risk index.

## Materials and Methods

### Participants and Procedure

The study protocol was reviewed and approved by the Human Subjects Research Committee at (Ben Gurion University) University. Data were collected from January 2018 to January 2019 through Prolific, an online research platform (Palan and Schitter, 2018). Mothers of children aged 17–19 months were initially approached via Prolific and invited to participate in the study. Mothers who were willing to participate signed online consent forms. The initial sample at T1 consisted of 207 mothers of children (*M* child age in months = 17.71, *SD* = 0.83; 60% male). Eight participants were excluded from the study due to child health or developmental problems (*n* = 3), maternal health problems (*n* = 4), or answering the attention-verifying items wrongly (“If you read this please mark 4”; *n* = 1). Thus, 199 participants comprised the final sample at T1. Demographic information is reported in Table 1. Participants were re-approached via Prolific 4 and 8 months later to participate at T2 (*n* = 149; *M* child age in months = 21.11, *SD* = 1.04) and T3 (*n* = 119; *M* child age in months = 25.21, *SD* = 1.04). Mothers were requested to complete a set of questionnaires at all three time points. Participants received 1.3 GBP for participating in T1 and 3 GBP for participating in T2 and T3.

**TABLE 1:** Sample demographic characteristics.

	*M*	*SD*	Range
Maternal age (years)	31.33	4.96	19–45
**Maternal education (percent)**			
> 12	1.5%		
Full high-school Diploma	50%		
Academic	48%		
**Current country or nationality (percent)**			
United Kingdom	79.2%		
United States	13.6%		
Europe	7.2%		
**Ethnicity (percent)**			
European White	92.5%		
African American	2.5%		
Asian	3.5%		
Other ethnicity	1.5%		
Number of children	1.84	0.95	1–6
**Family status (percent)**			
In a relationship or married	87%		
Separated or divorced	3.5%		
Single	9.5%		
**Employment status (percent)**			
Full-time	25.6%		
Part-time	44.7%		
Unemployed/homemaker	29.6%		

### Measures

#### Cumulative Media Use (CMU)

The CMU measure was constructed from four indicators that were selected based on the recommendations of the AAP for media use in early childhood (Council on Communications, and Media, 2016):

##### Child Average Daily Screen Time

Screen time was assessed using maternal report of average child screen time (i.e., watching television, watching videos/playing games on a handled device) during a typical weekday and weekend day. Weighted average scores for total screen time across time (weekdays and weekends) were calculated for all three time points. Screen time data at specific time points were not used for participants who reported aberrantly high child screen time (+ 2 *SD* above the mean) due to concerns regarding the reliability of these reports. These included nine participants at T1 (above 447.62 min per day), seven participants at T2 (above 379.62 min per day), and four participants at T3 (above 412.72 min a day).

##### Household Background Television

Mothers were asked to rate how often the television is on, if ever, in their household when someone is at home, even if no one is actually watching it, on a scale ranging from 0 (*Never*) to 5 (*Always*).

##### Use of Media to Regulate Child Distress

Mothers completed a version of the Coping with Toddlers’ Negative Emotion Scale (CTNES; Spinrad et al., 2007) that was modified for the current study. The CTNES consists of 12 different scenarios in which children exhibit distress (e.g., parent prohibits an activity). Mothers are asked to rate the likelihood to respond in seven different ways to children’s distress (i.e., distress reactions, minimizing the child’s distress, encouraging emotional expressiveness, punitive reactions, emotion focused, problem focused, and granting the child’s wish) that were rated on a scale ranging from 1 (*Very unlikely*) to 7 (*Very likely*). In the current study, four distress scenarios were presented to mothers to reduce participant burden, and an additional strategy was added: the likelihood of responding with the provision media to reduce the child’s distress (e.g., “If my child becomes angry because s/he is not allowed to have a snack when s/he wants it, I would offer to let my child play or watch something on my phone/tablet/computer/television”), which was the scale for the current variable. Items on this scale were averaged, and a higher score on this scale indicates a higher likelihood of using media to regulate child distress (α = 0.78, 0.79, and 0.80 for T1, T2, and T3, respectively).

##### Maternal Mobile Device Use

Mothers were asked to rate how often, if ever, they use media (for example, a mobile phone or tablet) to keep themselves occupied while spending time with their children on a scale ranging from 0 (*Never*) to 3 (*Often*).

##### Calculating the CMU Scores

CMU scores were calculated using a proportion-score approach (Moran et al., 2017). For each indicator, a proportion score is computed by dividing each individual score by the maximum score, yielding a proportion score with a maximum value of one. The composite score is then the mean of all proportion scores. This method is appropriate when risk factors are continuous, as it maintains the relative rank ordering of individuals, which is lost in dichotomization. Thus, this approach assumes that risk occurs on a continuum with varying degrees of severity (Ettekal et al., 2019).

For each time point, a CMU score was calculated by first dividing each individual risk indicator score by the maximum score within the current sample (yielding a proportion score with a maximum value of one) and then computing the mean of all four indicators to estimate a total score for each time point. Higher scores represent higher exposure to problematic media use. CMU scores ranged between 0.04 and 0.78 at T1, 0.08 and 0.83 at T2, and 0.12 and 0.76 at T3.

#### Child Focused Attention

Children’s focused attention abilities were measured using the Attentional Focusing subscale from the Early Childhood Behavior Questionnaire Short Form (ECBQ-SF; Putnam et al., 2006). The Attentional Focusing subscale includes six items that assess children’s ability to sustain duration of orienting on an object of attention and resist distractions (e.g., “When engaged in play with his/her favorite toy, how often did your child play for more than 10 min?”; “When engaged in an activity requiring attention, such as building with blocks, how often did your child move quickly to another activity?”; “While looking at picture books on his/her own, how often did your child become easily distracted?”). Mothers were asked to rate each item on a scale ranging from 1 (*Never*) to 7 (*Always*). Higher scores on this scale indicate better focused attention abilities (α = 0.70, 0.69, and 0.73 for T1, T2, and T3, respectively).

#### Covariates

Maternal education and child sex were included as covariates in all analyses, based on previous studies linking child media use to maternal education level (Vijakkhana et al., 2015) and indicating sex differences in attention abilities (Groot et al., 2004). Maternal education was rated on a scale from 1 (*Less than a high-school diploma*) to 6 (*Graduate degree*). In order to examine the unique role of media use above and beyond general parenting approaches, we also included two measures that reflect supportive and unsupportive parenting behaviors that were derived from the CTNES (Spinrad et al., 2007). The items on each scale of the CTNES were averaged to create the supportive (problem-focused, emotion-focused, and expressive encouragement; α = 0.83) and unsupportive (minimizing and punitive reaction; α = 0.76) subscales.

#### Missing Data and Attrition

Of the 199 participants who composed the final sample at T1, 149 participated in T2 and 119 at T3. No significant differences were found between participants who did not participate at T2 and T3 and those who participated at all three time points in maternal education level, child sex, and the study variables. In addition, participants who wrongly answered the attention-verifying items at T2 (*n* = 6) and T3 (*n* = 2) were excluded from those specific time points. To account for missing data, we utilized a full maximum likelihood (FIML) estimator for all analyses. FIML is well recognized as an effective method for analyzing longitudinal data with moderate to large amounts of missing data and has been demonstrated to provide less biased parameter estimates than other commonly used techniques, such as listwise deletion (Enders, 2013). Because FIML procedures allow for the use of all available data from each participant, the full sample of *n* = 199 was retained in all primary analyses.

#### Statistical Analysis

An autoregressive cross-lagged (ARCL) model was applied to test the main study hypothesis. The ARCL model represents a path model that simultaneously estimates the autoregressive relations (i.e., stability) of two or more variables that unfold over time, along with the cross-lagged relations between these variables (i.e., the time-lagged regressions across time points). The cross-lagged parameters are typically interpreted as the between-person effect of X at time 1 on Y at time 2, controlling for Y at time 1 (and vice versa). Thus, this model is particularly suitable for examining bidirectional relations between variables across several time points.

CMU and child-focused attention were estimated at all three time points. Autoregressive paths were specified within measurements of CMU and focused attention at T1, T2, and T3, and cross-lagged paths were specified between measures of CMU and focused attention across time points. Concurrent associations between variables within time points were estimated. All focal variables in the model were regressed on the selected covariates (i.e., maternal education, child sex, supportive and unsupportive parenting). Bootstrapping (with 10,000 resamples) was used to derive 95% confidence intervals for the direct and indirect effects. Model fit was determined using the root mean square error of approximation (RMSEA), standardized root mean square residual (SRMR), and comparative fit index (CFI). Adequate fit was defined as CFI values ≥ 0.95, RMSEA value ≤ 0.06, and SRMR values ≤ 0.08.

## Results

### Descriptive Statistics

Table 2 presents the bivariate correlations, means, and standard deviations for the study variables and covariates. Both the CMU and FA measures were significantly and positively correlated across time points. In addition, the CMU measures at all three time points were significantly negatively correlated with FA at T2 and T3. FA at T1 was also negatively correlated with CMU at T3. As for the study covariates, unsupportive parenting practices were positively linked with CMU at all-time points, and supportive parenting practices were negatively correlated with CMU at T1. In addition, maternal education was significantly negatively correlated with CMU at T2, and child sex was related to FA at T1 such that girls tended to have higher FA than boys.

**TABLE 2:** Unweighted means, standard deviations, and correlations among all study variables.

	1	2	3	4	5	6	7	8	9	10	11	12	13	14	15	16	17	18	19	20	21	22
1. CMU T1	–																					
2. CMU T2	0.71***	–																				
3. CMU T3	0.61***	0.63***	–																			
4. FA T1	–0.13	–0.13	−0.20*	–																		
5. FA T2	−0.30***	−0.37***	−0.41***	0.50***	–																	
6. FA T3	−0.18*	−0.34***	−0.35***	0.33***	0.59***	–																
7. ST T1	0.70***	0.53***	0.49***	–0.00	−0.17*	–0.10	–															
8. ST T2	0.36***	0.61***	0.34***	0.03	–0.15	−0.19*	0.49***	–														
9. ST T3	0.38***	0.46***	0.68***	–0.10	−0.23*	−0.20*	0.49***	0.62***	–													
10. BTV T1	0.48***	0.63***	0.46***	–0.02	−0.19*	–0.13	0.34***	0.29**	0.27**	–												
11. BTV T2	0.63***	0.55***	0.48***	–0.07	−0.22**	−0.20*	0.36***	0.34***	0.38***	0.64***	–											
12. BTV T3	0.41***	0.49***	0.62***	–0.03	−0.26**	–0.11	0.41***	0.31**	0.37***	0.73***	0.64***	–										
13. MREG T1	0.62***	0.42***	0.47***	–0.10	−0.25**	–0.11	0.41***	0.20*	0.36***	0.15	0.24**	0.13	–									
14. MREG T2	0.36***	0.52***	0.44***	−0.20*	−0.25**	−0.25**	0.34***	0.25**	0.43***	0.19*	0.23**	0.19*	0.61***	–								
15. MREG T3	0.40***	0.38***	0.58***	−0.21*	−0.35***	−0.39***	0.39***	0.19*	0.40***	0.15	0.25**	0.18	0.58***	0.63***	–							
16. PMU T1	0.63***	0.37***	0.18*	−0.15*	−0.16*	–0.06	0.11	–0.02	–0.11	0.20*	0.14*	0.01	0.10	–0.07	–0.06	–						
17. PMU T2	0.44***	0.58***	0.28**	–0.11	−0.28*	−0.22*	0.16	0.01	–0.06	0.10	0.13	–0.00	0.12	–0.02	0.03	0.60***	–					
18. PMU T3	0.30***	0.23*	0.54***	–0.12	–0.16	–0.15	–0.00	–0.12	0.04	0.02	–0.02	0.04	0.12	–0.05	–0.02	0.48***	0.56***	–				
19. MEDU	–0.05	−0.17*	–0.15	–0.08	0.05	0.11	–0.12	–0.08	−0.22*	−0.26**	−0.21**	−0.36***	0.05	−0.20*	–0.07	0.06*	0.07	0.18*	–			
20. Child sex	–0.02	0.00	–0.00	0.18*	0.05	0.02	–0.04	0.00	–0.01	0.04	0.09	0.06	0.05	0.08	0.08	–0.13	–0.09	–0.11	–0.04	–		
21. SUPP	−0.15*	0.01	–0.01	0.08	–0.03	–0.09	−0.20**	–0.12	−0.19*	0.05	–0.12	–0.03	–0.02	0.06	0.02	–0.06	0.02	0.12	0.06	0.12	–	
22. UNSUPP	0.25***	0.21*	0.25**	–0.04	–0.15	–0.13	0.31***	0.19*	0.22*	0.11	0.18*	0.15	0.26***	0.22**	0.27**	–0.04	0.00	0.00	–0.10	0.06	–0.06	–
Mean	0.40	0.41	0.41	4.25	4.42	4.73	132.40	131.22	150.33	2.41	2.45	2.49	2.62	2.73	2.97	1.28	1.18	1.12	NA	NA	5.16	3.19
*SD*	0.15	0.14	0.13	0.96	0.87	0.83	100.64	80.05	83.35	1.08	1.05	1.09	1.32	1.35	1.44	0.91	0.94	0.84	NA	NA	0.85	1.17

T1, age 18 months; T2, age 22 months; T3, age 26 months; CMU, cumulative media use; FA, focused attention; ST, screen time; BTV, background television; MREG, use of media to regulate child distress; PMU, maternal mobile device use; MEDU, maternal education; SUPP, supportive parenting; UNSUPP, unsupportive parenting; *p < 0.05, **p < 0.01, ***p < 0.001.

### ARCL Model: Longitudinal Links Between CMU and FA

**FIGURE 1:**
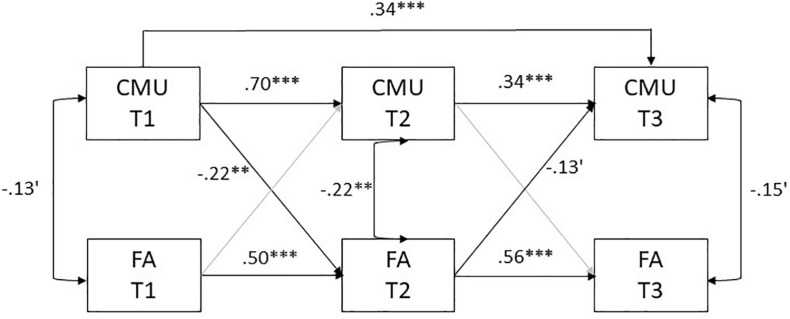
An ARCL model estimating autoregressive and cross-lagged paths between repeated measures of CMU and FA between T1 to T3. Notes: For ease of presentation only significant paths are included in the figure; The following covariates were included in the model, but are not depicted in this figure: maternal education, child sex, supportive and unsupportive parenting behaviors; CVTU, cumulative media use; FA, focused attention; ′*p* < 0.1, ^∗∗^*p* < 0.01, ^∗∗∗^*p* < 0.001.

We first estimated a model in which autoregressive paths were specified within measurements of CMU and FA and cross-lagged paths were specified between measures of CMU and FA across the three time points. In addition, concurrent associations between variables within time points were estimated, and all focal variables in the model were regressed on the selected covariates. However, model fit was unsatisfactory, χ^2^(4) = 21.99, *p* = 0.01, CFI = 0.96, RMSEA = 0.07, SRMR = 0.03. Analysis of modification indices suggested that the addition of a path between CMU at T1 and CMU at T3 would improve model fit. Thus, this path was added to the final model (Figure 1). Path coefficients remained similar to the previous model, and model fit was improved: χ^2^(3) = 9.28, *p* = 0.41, CFI = 0.99, RMSEA = 0.01, SRMR = 0.02.

All autoregressive paths were significant, indicating stability in CMU and FA over time. In addition, CMU at T1 negatively predicted FA at T2 (β = −0.22, *p* = 0.001, [95% CI, −0.35 to −0.08]), indicating that higher exposure to CMU was longitudinally related to lower FA. Moreover, there was a significant negative indirect path between CMU at T1 and FA at T3 via FA at T2 (β = −0.12, *p* = 0.003, [95% CI, −0.20 to −0.04]). Notably, the path between FA at T2 and CMU at T3 showed a non-significant trend (β = −0.13, *p* = 0.079, [95% CI, −0.28 to 0.01]).

### Testing the Predictive Efficacy of the CMU Measure

We first examined whether the singular factors that composed the CMU score were predictive of FA. To that aim, we estimated an ARCL model in which autoregressive and crossed-lagged paths were specified within and between measurements of child screen time, background television, use of media to regulate distress, maternal mobile device use, and FA across the three time points. All focal variables in the model were also regressed on the selected covariates. None of the singular variables significantly predicted FA at T2 and T3 (see Table 3).

**TABLE 3:** Standardized path coefficients for the ARCL model with the singular CMU factors.

	Estimate	SE	*p*-value
ST T1 → FA T2	−0.00	0.09	0.974
MREG T1 → FA T2	−0.12	0.07	0.111
BTV T1 → FA T2	−01	0.08	0.106
PMU T1 → FA T2	0.07	0.06	0.273
SUPP → FA T2	−0.05	0.07	0.455
UNSUPP → FA T2	−0.07	0.07	0.315
FA T1 → FA T2	0.48	0.06	0.000
Maternal education → FA T2	0.08	0.07	0.220
Child sex → FA T2	0.04	0.07	0.542
ST T1 → ST T2	0.43	0.03	0.000
MREG T1 → ST T2	−0.03	0.03	0.702
BTV T1 → ST T2	0.17	0.04	0.037
PMU T1 → ST T2	−0.07	0.04	0.295
SUPP → ST T2	−0.03	0.03	0.649
UNSUPP → ST T2	0.07	0.03	0.322
FA T1 → ST T2	−0.01	0.03	0.850
Maternal education → ST T2	0.04	0.03	0.898
Child sex → ST T2	0.04	0.07	0.542
ST T1 → MREG T2	0.08	0.08	0.280
MREG T1 → MREG T2	0.56	0.06	0.000
BTV T1 → MREG T2	0.01	0.07	0.802
PMU T1 → MREG T2	−0.11	0.06	0.061
SUPP → MREG T2	0.08	0.06	0.205
UNSUPP → MREG T2	0.02	0.06	0.690
FA T1 → MREG T2	−0.11	0.06	0.008
Maternal education → MREG T2	−0.23	0.06	0.000
Child sex → MREG T2	0.08	0.06	0.192
ST T1 → BTV T2	0.04	0.08	0.617
MREG T1 →BTV T2	0.00	0.07	0.967
BTV T1 → BTV T2	0.59	0.06	0.000
PMU T1 → BTV T2	0.09	0.06	0.132
SUPP → BTV T2	0.17	0.06	0.007
UNSUPP → BTV T2	−0.01	0.06	0.852
FA T1 → BTV T2	0.02	0.06	0.726
Maternal education → BTV T2	−0.14	0.06	0.036
Child sex → BTV T2	0.01	0.06	0.856
ST T1 → PMU T2	0.16	0.08	0.059
MREG T1 → PMU T2	0.00	0.07	0.992
BTV T1 → PMU T2	−0.02	0.07	0.717
PMU T1 → PMU T2	0.57	0.05	0.000
SUPP → PMU T2	0.05	0.07	0.413
UNSUPP → PMU T2	−0.02	0.07	0.765
FA T1 → PMU T2	−0.05	0.07	0.437
Maternal education → BTV T2	0.05	0.07	0.462
Child sex → BTV T2	−0.02	0.06	0.774
ST T2 → FA T3	−0.08	0.08	0.333
MREG T2 → FA T3	−0.03	0.08	0.703
BTV T2 → FA T3	0.04	0.08	0.591
PMU T2 → FA T3	−0.03	0.08	0.663
FA T2 → FA T3	0.51	0.08	0.000
SUPP → FA T3	−0.06	0.07	0.389
UNSUPP → FA T3	−0.03	0.07	0.610
FA T1 → FA T3	0.09	0.09	0.297
Maternal education → FA T3	0.09	0.08	0.238
Child sex → FA T3	0.00	0.07	0.933
ST T2 → ST T3	0.52	0.07	0.000
MREG T2 → ST T3	0.24	0.07	0.002
BTV T2 → ST T3	0.00	0.07	0.906
PMU T2 → ST T3	−0.04	0.07	0.564
FA T2 → ST T3	−0.05	0.08	0.521
SUPP → ST T3	−0.12	0.06	0.080
UNSUPP → ST T3	0.03	0.06	0.628
FA T1 → ST T3	−0.04	0.08	0.563
Maternal education → ST T3	−0.10	0.07	0.151
Child sex → ST T3	−0.06	0.06	0.364
ST T2 → MREG T3	0.07	0.07	0.831
MREG T2 → MREG T3	0.38	0.09	0.000
BTV T2 → MREG T3	−0.07	0.07	0.306
PMU T2 → MREG T3	−0.06	0.07	0.359
FA T2 → MREG T3	−0.17	0.08	0.037
SUPP → MREG T3	0.00	0.07	0.987
UNSUPP → MREG T3	0.07	0.07	0.280
FA T1 → MREG T3	−0.03	0.08	0.656
Maternal education → MREG T3	−0.04	0.07	0.540
Child sex → MREG T3	0.02	0.06	0.739
MREG T1 → MREG T3	0.29	0.08	0.001
ST T2 → BTV T3	0.06	0.07	0.355
MREG T2 → BTV T3	−0.08	0.07	0.204
BTV T2 → BTV T3	0.50	0.07	0.000
PMU T2 → BTV T3	−0.04	0.06	0.540
FA T2 → BTV T3	−0.06	0.07	0.428
SUPP → BTV T3	0.00	0.06	0.951
UNSUPP → BTV T3	0.05	0.06	0.370
FA T1 → BTV T3	−0.03	0.07	0.661
Maternal education → BTV T3	−0.12	0.06	0.072
Child sex → BTV T3	0.02	0.05	0.678
BTV T1 → BTV T3	0.27	0.07	0.000
ST T2 → PMU T3	−0.10	0.09	0.244
MREG T2 → PMU T3	−0.02	0.09	0.775
BTV T2 → PMU T3	0.09	0.08	0.787
PMU T2 → PMU T3	0.54	0.07	0.000
FA T2 → PMU T3	−0.01	0.09	0.910
SUPP → PMU T3	0.03	0.08	0.666
UNSUPP → PMU T3	0.04	0.07	0.602
FA T1 → PMU T3	0.01	0.09	0.860
Maternal education → PMU T3	0.14	0.08	0.081
Child sex → PMU T3	−0.07	0.07	0.341

**Model fit: χ^2^(18) = 26.72, p = 0.08, CFI = 0.98, RMSEA = 0.04, SRMR = 0.02; T1, age 18 months; T2, age 22 months; T3, age 26 months; CMU, cumulative media use; FA, focused attention; ST, screen time; BTV, background television; MREG, use of media to regulate child distress; PMU, maternal mobile device use; MEDU, maternal education; SUPP, supportive parenting; UNSUPP, unsupportive parenting.**

We next analyzed the efficacy of the CMU score at T1 in predicting FA at T2 compared to each of the singular factors that composed the CMU. We estimated four models, each including the original ARCL model with the addition of one of the individual factors (i.e., child screen time, background television, use of media to regulate distress, and maternal mobile device use) at all three time points. In all four models, while T1 CMU was a significant predictor of T2 FA, the singular factors were not (see Supplementary Figures 1–4).

## Discussion

The goal of the current study was to develop a cumulative media use index that includes multiple aspects of young children’s direct and indirect media use (CMU) and examine its predictive associations with children’s later focused attention abilities. Consistent with our hypothesis, higher levels of CMU predicted lower consecutive attention abilities during toddlerhood. Moreover, the CMU score appeared to be a better predictor of attention abilities than any of the singular measures of media use. Our findings demonstrate the possible implications that extensive media use may have for children’s focused attention abilities and indicate the importance of including multiple contextual factors of media use in studies of media and child development.

Previous research on the link between media use and attention in early childhood is limited in three main ways. First, although there is some evidence that excessive screen viewing time in early childhood predicts subsequent attention problems (Christakis et al., 2004; Cheng et al., 2010), these studies assessed attention abilities only as an outcome, precluding the ability to consider the bidirectional links between media use and attention. While it is possible that excessive exposure to screen media interferes with the development of attention skills, it is also plausible that children with limited attention spans are more drawn to screen media, and as a result parents often expose them to screens to occupy or soothe them (Kostyrka-Allchorne et al., 2017). Second, previous research did not consider the broader family context of children’s exposure to media, such as how the media are used by all member of the household, including children’s direct and indirect exposures (Barr et al., 2010). Addressing these contextual factors is particularly important in early childhood because during this period children’s self-regulatory and attention abilities are limited, and the home environment plays a vital role in fostering these emerging abilities (Kopp, 1989). Finally, the majority of previous research focused on attention abilities (e.g., EF) or attention problems in the preschool period, and therefore little is known about the potential impact of screen media in early childhood.

To our knowledge, this study is the first to address these aforementioned limitations by applying a repeated-measure longitudinal design and examining media use from an ecological perspective that includes, in addition to direct media exposure, indirect exposure to media and media use practices. Drawing from the cumulative risk literature (Evans et al., 2013), we created a cumulative media use index that included four aspects of media use. Results show that higher CMU at age 18 months directly predicted lower FA at age 22 months. In addition, CMU at 18 months indirectly predicted lower FA at age 26 months via FA at 22 months. However, CMU at 22 months was not a significant predictor of FA at 26 months. These findings suggest that elevated media use in early toddlerhood (age 18 months) can initiate a cascade of attention difficulties that persist across toddlerhood.

Why would exposure to media at 18 months of age be critical for the development of FA? The attentional network framework (Posner et al., 2014) suggests that the orienting network exerts much of the control over other attention networks during infancy and toddlerhood, while the executive attention network becomes increasingly dominant during the second year of life. The time period between 18 and 24 months represents a developmental period in which both of these attention systems are still developing rapidly. After age 24 months, the orienting system reaches a plateau and individual differences in orienting abilities stabilize (Posner et al., 2014). Thus, 18 months may be a time period in which children are particularly susceptible to environmental experiences, such as excessive media use, that support or hinder the development of both the orienting and the executive attention networks.

Contrary to previous research on media use and child outcomes (Magee et al., 2014; Kostyrka-Allchorne et al., 2017; Cliff et al., 2018), we only found unidirectional paths between CMU and FA, with the reverse associations being non-significant. Although there was a negative link between FA at 22 and CMU at 26 months, this path did not reach significance (*p* = 0.08) and therefore cannot be interpreted. These discrepant findings may be related to the different age groups between samples. Two studies that found significant links between children’s self-regulation and sleep and consecutive media use used samples of 4–6-years-old children (Magee et al., 2014; Cliff et al., 2018), who are able to use media independently, whereas in our younger sample (ages 18–26 months) media use may be mainly determined by parents and less driven by child characteristics. It is also possible that the current study did not have sufficient statistical power to detect small effect sizes due to our modest sample size.

To our knowledge, this study is the first to apply the cumulative risk approach to media exposure. Thus, an additional goal of this study was to examine the predictive utility of the CMU index compared to the singular aspects of media use. Our results indicate that CMU at age 18 months was a better predictor of FA at 22 than any of the singular measures. This finding coincides with the cumulative risk literature that has consistently demonstrated that children exposed to cumulative risk factors in early life show more adverse outcomes than those exposed to singular risk factors (Evans et al., 2013). The CMU index may confer increased risk for attention problems because it exerts continuous interference to the attentional system spanning the child’s day, rather than segmented periods of interference, such as daily screen viewing time. Children with high CMU are at risk for experiencing distractions in toy play and social interactions caused by background television and parental mobile phone use, as well as increased arousal and difficulties in regulating attention due to parental use of media to regulate their distress. Moreover, elevated screen viewing time often includes prolonged exposure to fast-paced content that is hypothesized to prompt a scanning–shifting attentional style that may hinder the ability to focus attention in natural settings such as toy play (Nikkelen et al., 2014). Cumulative exposure to these distracting and arousing experiences throughout the day also denies children opportunities to participate in environmental experiences that are crucial for fostering their emerging FA skills, such as contingent social interactions, mutual joint attention during play, and parent–child reading interactions (Zimmerman and Christakis, 2007). Moreover, a recent study suggests that increased use of screen-based media (as measured by access to screens, frequency of use, content, and co-viewing) may alter children’s cognitive abilities through neural pathways, such as decreased microstructural integrity of the brain white matter tracts that support language, executive functions, and language abilities (Hutton et al., 2020a).

The CMU index may tap into general parenting practices, and there is therefore reason to suspect that the link between CMU and FA is actually driven by the link between parenting practices and CMU. Children’s screen-based media use has been previously correlated with less stimulating home cognitive environments, and higher use of authoritarian and permissive parenting styles (Howe et al., 2017; Hutton et al., 2020b). Indeed, consistent with previous literature, in the current study unsupportive parenting practices were positively related with the CMU index, implying that children of mothers who frequently use parenting practices such as punishment and minimizing children’s distress may also be exposed to multiple aspects of media use in the household. However, the CMU index was a significant predictor of children’s FA even when controlling for both supportive and unsupportive parenting practices. These findings highlight the unique implications of media use for children’s attention abilities, beyond the potential contribution of general parenting practices.

## Limitations

The findings of the current study should be considered in light of several limitations. First, our assessment of media exposure did not include the type of content (e.g., fast/slow-paced, entertainment/educational) that children are exposed to. There is evidence that the links between children’s overall screen time and attention problems are only evident when watching entertainment or adult-directed content, but not when watching educational content (Zimmerman and Christakis, 2007; Barr et al., 2010; Kostyrka-Allchorne et al., 2017). In fact, viewing educational media content was linked to increased EFs in children at high demographic risk (Linebarger et al., 2014). Second, our indicators of media use and attention are based exclusively on maternal reports, which may result in report bias or inaccurate estimates. Applying a multi-method assessment of media use that also includes daily time-use diaries and passive sensing applications that detect media use on mobile devices can reduce parents’ report bias and yield more accurate estimates (Barr, 2019). Similarly, using observational tasks of children’s FA abilities in naturalistic setting such as toy play (Lansink et al., 2000) could further increase measurement validity. Finally, the correlational nature of this study precludes the inference of causal relations between media use and attention skills. Because our focus was on cumulative exposure to media and the examination of associations over time, it is not possible to examine our research questions in a controlled experimental design. However, an important next step could be to examine the immediate impact of exposure to increasing levels of our four media use indictors on children’s attention abilities in an experimental design (e.g., Lillard et al., 2015).

## Conclusion

Our findings demonstrate that elevated exposure to media predicts lower subsequent focused attention abilities during toddlerhood. In this study, we addressed two key limitations of previous research by applying a repeated-measure longitudinal design that considers concurrent and cross-lagged associations between media use and attention, and by broadening the measurement of media use from the *amount* of direct exposure to include contextual factors reflecting *how* the media are used in the household. Our work adds to the extant literature by documenting that a broad and cumulative approach to assess media use is effective for understanding the potential implications of media use on children’s cognitive development.

The findings of this study can inform family-based prevention initiatives designed to promote balanced household media use. Increasing parental awareness of the possible implications of indirect media use such as background television, parental mobile phone use, and the use of media to regulate distress, along with encouraging “media-free” time slots and the use of alternative regulatory strategies, can help families use media in a thoughtful and appropriate manner.

## Data Availability Statement

The datasets presented in this article are not readily available because the ethics approval does not permit sharing the dataset. Requests to access the datasets should be directed to NG-S, gueron@post.bgu.ac.il.

## Ethics Statement

The studies involving human participants were reviewed and approved by Human Subjects Research Committee, Ben-Gurion University of the Negev. The patients/participants provided their written informed consent to participate in this study.

## Author Contributions

NG-S designed and conceptualized the study, conceived the ideas for the manuscript, and performed data analysis and interpretation. AG-H designed and conceptualized the study, performed data collection and analysis, and provided revisions to scientific content of the manuscript. Both authors contributed to the article and approved the submitted version.

## Conflict of Interest

The authors declare that the research was conducted in the absence of any commercial or financial relationships that could be construed as a potential conflict of interest.

## References

[B1] Anderson D. R. Evans M. K. (2001). Peril and potential of media for infants and toddlers. *Zero Three* 22 10–16.

[B2] Anderson D. R. Pempek T. A. (2005). Television and very young children. *Am. Behav. Sci.* 48 505–522. 10.1177/0002764204271506

[B3] Anderson D. R. Subrahmanyam K. (2017). Digital screen media and cognitive development. *Pediatrics* 140 S57–S61. 10.1542/peds.2016-1758C 29093033

[B4] Barr R. (2019). growing up in the digital age: early learning and family media ecology. *Curr. Dir. Psychol. Sci.* 28 341–346. 10.1177/0963721419838245 31423053 PMC6697422

[B5] Barr R. Lauricella A. Zack E. Calvert S. L. (2010). Infant and early childhood exposure to adult-directed and child-directed television programming: relations with cognitive skills at age four. *Merrill Palmer Q.* 56 21–48. 10.1353/mpq.0.0038

[B6] Blankson A. N. O’Brien M. Leerkes E. M. Calkins S. D. Marcovitch S. (2015). Do hours spent viewing television at ages 3 and 4 predict vocabulary and executive functioning at age 5? *Merrill Palmer Q.* 61 264–289. 10.13110/merrpalmquar1982.61.2.0264

[B7] Cheng S. Maeda T. Yoichi S. Yamagata Z. Tomiwa K. (2010). Early television exposure and children’s behavioral and social outcomes at age 30 months. *J. Epidemiol.* 20(Suppl.2), 1–2. 10.2188/jea.JE20090179 20179364 PMC3920399

[B8] Christakis D. A. Zimmerman F. J. DiGiuseppe D. L. McCarty C. A. (2004). Early television exposure and subsequent attentional problems in children. *Pediatrics* 113 708–713. 10.1542/peds.113.4.708 15060216

[B9] Cliff D. P. Howard S. J. Radesky J. S. McNeill J. Vella S. A. (2018). Early childhood media exposure and self-regulation: bidirectional longitudinal associations. *Acad. Pediatr.* 18 813–819. 10.1016/j.acap.2018.04.012 29704999

[B10] Colombo J. Cheatham C. (2007). “The emergence of endogenous attention in infancy and early childhoodNo Title,” in *Advances in Child Development and Behavior*, ed. Kail R., (New York, NY: Elsevier), 283–322. 10.1016/s0065-2407(06)80010-8 17120808

[B11] Colombo J. Salley B. (2015). “Biopsychosocial perspectives on the development of attention in infancy,” in *Handbook of Infant Biopsychosocial Development*, ed. Calkins S. D., (New York, NY: Guilford Press), 71–96.

[B12] Comas M. Valentino K. Borkowski J. G. (2014). Maternal depressive symptoms and child temperament: longitudinal associations with executive functioning. *J. Appl. Dev. Psychol.* 35 156–167. 10.1016/j.appdev.2014.03.005

[B13] Council on Communications, and Media (2016). Media and young minds. *Pediatrics* 138:e20162591. 10.1542/peds.2016-2591 27940793

[B14] Courage M. L. (2017). “Screen media and the youngest viewers: implications for attention and learning,” in *Cognitive Development in Digital Contexts*, eds Blumberg F. C. Brooks P. J., (Amsterdam: Elsevier Inc), 10.1016/B978-0-12-809481-5.00001-8

[B15] Enders C. K. (2013). Dealing with missing data in developmental research. *Child Dev. Perspect.* 7 27–31. 10.1111/cdep.12008

[B16] Ettekal I. Eiden R. D. Nickerson A. B. Schuetze P. (2019). Comparing alternative methods of measuring cumulative risk based on multiple risk indicators: are there differential effects on children’s externalizing problems? *PLoS One* 14:e0219134. 10.1371/journal.pone.0219134 31269048 PMC6609027

[B17] Evans G. W. Li D. Whipple S. S. (2013). Cumulative risk and child development. *Psychol. Bull.* 139 1342–1396. 10.1037/a0031808 23566018

[B18] Foster E. M. Watkins S. (2010). The value of reanalysis: TV viewing and attention problems. *Child Dev.* 81 368–375. 10.1111/j.1467-8624.2009.01400.x 20331673

[B19] Gach E. J. Ip K. I. Sameroff A. J. Olson S. L. (2018). Early cumulative risk predicts externalizing behavior at age 10: the mediating role of adverse parenting. *J. Fam. Psychol.* 32 92–102. 10.1037/fam0000360 29543487

[B20] Garon N. Bryson S. E. Smith I. M. (2008). Executive function in preschoolers: a review using an integrative framework. *Psychol. Bull.* 134 31–60. 10.1037/0033-2909.134.1.31 18193994

[B21] Gordon-Hacker A. Gueron-Sela N. (2020). Maternal use of media to regulate child distress: a double-edged sword? Longitudinal links to toddlers’ negative emotionality. *Cyberpsychol. Behav. Soc. Network.* 23 400–405. 10.1089/cyber.2019.0487 32345033

[B22] Groot A. S. de Sonneville L. M. J. Stins J. F. Boomsma D. I. (2004). Familial influences on sustained attention and inhibition in preschoolers. *J. Child Psychol. Psychiatry Allied Disciplines* 45 306–314. 10.1111/j.1469-7610.2004.00222.x 14982244

[B23] Gueron-Sela N. Camerota M. Willoughby M. T. Vernon-Feagans L. Cox M. J. (2018). Maternal depressive symptoms, mother-child interactions, and children’s executive function. *Dev. Psychol.* 54 71–82. 10.1037/dev0000389 28933882 PMC5750080

[B24] Hiniker A. Sobel K. Suh H. Sung Y. C. Lee C. P. Kientz J. A. (2015). “Texting while parenting: how adults use mobile phones while caring for children at the playground,” in *Conference on Human Factors in Computing Systems - Proceedings*, Seoul, 727–736. 10.1145/2702123.2702199

[B25] Howe A. S. Heath A. L. M. Lawrence J. Galland B. C. Gray A. R. Taylor B. J. (2017). Parenting style and family type, but not child temperament, are associated with television viewing time in children at two years of age. *PLoS One* 12:e0188558. 10.1371/journal.pone.0188558 29261676 PMC5737952

[B26] Hutton J. S. Dudley J. Horowitz-Kraus T. Dewitt T. Holland S. K. (2020a). Associations between screen-based media use and brain white matter integrity in preschool-aged children. *JAMA Pediatr.* 174 1–10. 10.1001/jamapediatrics.2019.3869 31682712 PMC6830442

[B27] Hutton J. S. Huang G. Sahay R. D. DeWitt T. Ittenbach R. F. (2020b). A novel, composite measure of screen-based media use in young children (ScreenQ) and associations with parenting practices and cognitive abilities. *Pediatr. Res.* 87 1211–1218. 10.1038/s41390-020-0765-1 32050256

[B28] Johansson M. Marciszko C. Gredebäck G. Nyström P. Bohlin G. (2015). Sustained attention in infancy as a longitudinal predictor of self-regulatory functions. *Infant Behav. Dev.* 41 1–11. 10.1016/j.infbeh.2015.07.001 26241679

[B29] Kabali H. K. Irigoyen M. M. Nunez-Davis R. Budacki J. G. Mohanty S. H. Leister K. P. (2015). Exposure and use of mobile media devices by young children. *Pediatrics* 136 1044–1050. 10.1542/peds.2015-2151 26527548

[B30] Kannass K. N. Oakes L. M. Shaddy D. J. (2006). A longitudinal investigation of the development of attention and distractibility. *J. Cogn. Dev.* 7 381–409. 10.1207/s15327647jcd0703_8

[B31] Kildare C. A. Middlemiss W. (2017). Impact of parents mobile device use on parent-child interaction: a literature review. *Comput. Hum. Behav.* 75 579–593. 10.1016/j.chb.2017.06.003

[B32] Kirkorian H. L. Pempek T. A. Murphy L. A. Schmidt M. E. Anderson D. R. (2009). The impact of background television on parent-child interaction. *Child Dev.* 80 1350–1359. 10.1111/j.1467-8624.2009.01337.x 19765004

[B33] Kopp C. B. (1989). Regulation of distress and negative emotions: a developmental view. *Dev. Psychol.* 25 343–354. 10.1037/0012-1649.25.3.343

[B34] Kostyrka-Allchorne K. Cooper N. R. Simpson A. (2017). The relationship between television exposure and children’s cognition and behaviour: a systematic review. *Dev. Rev.* 44 19–58. 10.1016/j.dr.2016.12.002

[B35] Landhuis C. E. Poulton R. Welch D. Hancox R. J. (2007). Does childhood television viewing lead to attention problems in adolescence? Results from a prospective longitudinal study. *Pediatrics* 120 532–537. 10.1542/peds.2007-0978 17766526

[B36] Lansink J. M. Mintz S. Richards J. E. (2000). The distribution of infant attention during object examination. *Dev. Sci.* 3 163–170. 10.1111/1467-7687.00109

[B37] Lawson K. R. Ruff H. A. (2004). Early focused attention predicts outcome for children born prematurely. *J. Dev. Behav. Pediat.* 25 399–406. 10.1097/00004703-200412000-00003 15613988

[B38] Lillard A. S. Drell M. B. Richey E. M. Boguszewski K. Smith E. D. (2015). Further examination of the immediate impact of television on children’s executive function. *Dev. Psychol.* 51 792–805. 10.1037/a0039097 25822897

[B39] Linebarger D. L. Barr R. Lapierre M. A. Piotrowski J. T. (2014). Associations between parenting, media use, cumulative risk, and children’s executive functioning. *J. Dev. Behav. Pediatr.* 35 367–377. 10.1097/dbp.0000000000000069 25007059

[B40] Magee C. A. Lee J. K. Vella S. A. (2014). Bidirectional relationships between sleep duration and screen time in early childhood. *JAMA Pediatr.* 168 465–470. 10.1001/jamapediatrics.2013.4183 24589672

[B41] Miller M. Iosif A. M. Young G. S. Hill M. M. Ozonoff S. (2018). Early detection of ADHD: insights from infant siblings of children with autism. *J. Clin. Child Adolesc. Psychol.* 47 737–744. 10.1080/15374416.2016.1220314 27732091 PMC5436956

[B42] Moran L. Lengua L. J. Zalewski M. Ruberry E. Klein M. Thompson S. Kiff C. (2017). Variable- and person-centered approaches to examining temperament vulnerability and resilience to the effects of contextual risk. *J. Res. Personal*. 67, 61–74. 10.1016/j.jrp.2016.03.003 28408769 PMC5386509

[B43] Myruski S. Gulyayeva O. Birk S. Pérez-Edgar K. Buss K. A. Dennis-Tiwary T. A. (2018). Digital disruption? Maternal mobile device use is related to infant social-emotional functioning. *Dev. Sci.* 21 1–9. 10.1111/desc.12610 28944600 PMC5866735

[B44] Nathanson A. I. Aladé F. Sharp M. L. Rasmussen E. E. Christy K. (2014). The relation between television exposure and executive function among preschoolers. *Dev. Psychol.* 50 1497–1506. 10.1037/a0035714 24447117

[B45] Nikkelen S. W. C. Valkenburg P. M. Huizinga M. Bushman B. J. (2014). Media use and ADHD-related behaviors in children and adolescents: a meta-analysis. *Dev. Psychol.* 50 2228–2241. 10.1037/a0037318 24999762

[B46] Palan S. Schitter C. (2018). Prolific.ac—A subject pool for online experiments. *J. Behav. Exp. Finance* 17 22–27. 10.1016/j.jbef.2017.12.004

[B47] Posner M. I. Rothbart M. K. Sheese B. E. Voelker P. (2014). Developing attention: behavioral and brain mechanisms. *Adv. Neurosci.* 2014:405094. 10.1155/2014/405094 25110757 PMC4125572

[B48] Putnam S. P. Gartstein M. A. Rothbart M. K. (2006). Measurement of fine-grained aspects of toddler temperament: the early childhood behavior questionnaire. *Infant Behav. Dev.* 29 386–401. 10.1016/j.infbeh.2006.01.004 17138293 PMC4334385

[B49] Radesky J. Miller A. L. Rosenblum K. L. Appugliese D. Kaciroti N. Lumeng J. C. (2015). Maternal mobile device use during a structured parent–child interaction task. *Acad. Pediatr.* 15 238–244. 10.1016/j.acap.2014.10.001 25454369 PMC4355325

[B50] Radesky J. S. Kistin C. J. Zuckerman B. Nitzberg K. Gross J. Kaplan-Sanoff M. (2014a). Patterns of mobile device use by caregivers and children during meals in fast food restaurants. *Pediatrics* 133 e843–e849. 10.1542/peds.2013-3703 24616357

[B51] Radesky J. S. Silverstein M. Zuckerman B. Christakis D. A. (2014b). Infant self-regulation and early childhood media exposure. *Pediatrics* 133 e1172–e1178. 10.1542/peds.2013-2367 24733868 PMC4006432

[B52] Radesky J. S. Peacock-Chambers E. Zuckerman B. Silverstein M. (2016). Use of mobile technology to calm upset children: associations with social-emotional development. *JAMA Pediatr.* 170 397–399. 10.1001/jamapediatrics.2015.4260 26928293

[B53] Rideout V. (2017). *The Common Sense Census: Media use by kids Age Zero to Eight.* San Francisco, CA: Common Sense Media.

[B54] Ruff H. A. Capozzoli M. C. (2003). Development of attention and distractibility in the first 4 years of life. *Dev. Psychol.* 39 877–890. 10.1037/0012-1649.39.5.877 12952400

[B55] Ruff H. A. Lawson K. R. (1990). Development of sustained, focused attention in \bung. *Child. During Free Play* 26 85–93. 10.1037/0012-1649.26.1.85

[B56] Ruff H. A. Rothbart M. K. (1996). *Attention in Early Development: Themes and Variations.* New York, NY: Oxford University Press.

[B57] Sameroff A. (2010). A unified theory of development: a dialectic integration of nature and nurture. *Child Dev.* 81 6–22. 10.1111/j.1467-8624.2009.01378.x 20331651

[B58] Schmidt M. E. Pempek T. A. Kirkorian H. L. Lund A. F. Anderson D. R. (2008). The effects of background television on the toy play behavior of very young children. *Child Dev.* 79 1137–1151. 10.1111/j.1467-8624.2008.01180.x 18717911

[B59] Setliff A. E. Courage M. L. (2011). Background television and infants’ allocation of their attention during toy play. *Infancy* 16 611–639. 10.1111/j.1532-7078.2011.00070.x 32693498

[B60] Spinrad T. L. Eisenberg N. Gaertner B. Popp T. Smith C. L. Kupfer A. (2007). Relations of maternal socialization and toddlers’ effortful control to children’s adjustment and social competence. *Dev. Psychol.* 43 1170–1186. 10.1037/0012-1649.43.5.1170 17723043 PMC2096418

[B61] Stevens T. Barnard-Brak L. To Y. (2009). Television viewing and symptoms of inattention and hyperactivity across time: the importance of research questions. *J. Early Intervent.* 31 215–226. 10.1177/1053815109338562

[B62] Tamana S. K. Ezeugwu V. Chikuma J. Lefebvre D. L. Azad M. B. Moraes T. J. (2019). Screen-time is associated with inattention problems in preschoolers: results from the CHILD birth cohort study. *PLoS One* 14:e0213995. 10.1371/journal.pone.0213995 30995220 PMC6469768

[B63] Vijakkhana N. Wilaisakditipakorn T. Ruedeekhajorn K. Pruksananonda C. Chonchaiya W. (2015). Evening media exposure reduces night-time sleep. *Acta Paediat. Int. J. Paediatr.* 104 306–312. 10.1111/apa.12904 25521612

[B64] Yu C. Smith L. B. (2016). The social origins of sustained attention in one-year-old human infants. *Curr. Biol.* 26 1235–1240. 10.1016/j.cub.2016.03.026 27133869 PMC5387765

[B65] Zimmerman F. J. Christakis D. A. (2007). Associations between content types of early media exposure and subsequent attentional problems. *Pediatrics* 120 986–992. 10.1542/peds.2006-3322 17974735

