# Supplementary material for: Longitudinal Links Between Media Use and Focused Attention Through Toddlerhood: A Cumulative Risk Approach
Source: Front Psychol. 2020 Nov 2;11:569222. doi: 10.3389/fpsyg.2020.569222 (PMC7667257; doi:10.3389/fpsyg.2020.569222)
Supplement: Supplementary file 2 [file Image_2.pdf]

Supplementary Figure 2.

An ARCL model estimating autoregressive and cross-lagged paths between repeated measures of CMU, MREG and FA between T1 to T3.

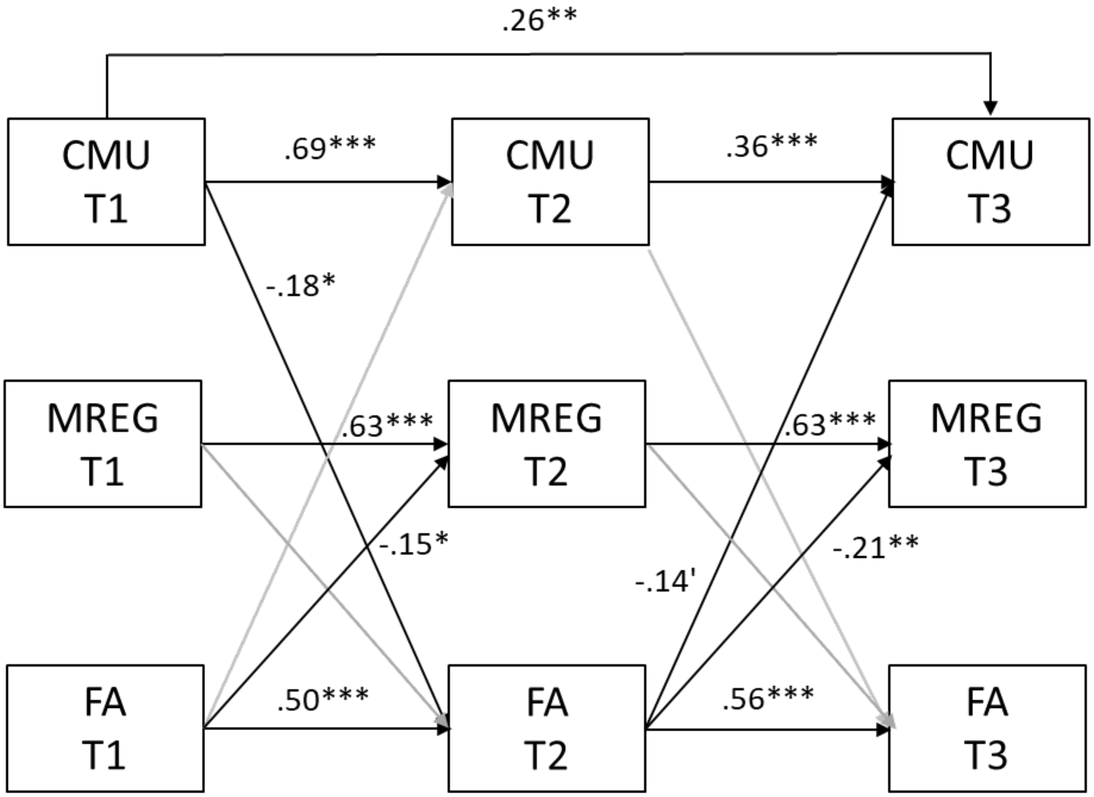

Note. For ease of presentation only significant paths are included in the figure, and all concurrent associations within time points were estimated but are not presented; The following covariates were included in the model, but are not depicted in this figure: maternal education, child sex, supportive and unsupportive parenting behaviors; CMU = cumulative media use, MREG = use of media to regulate child distress, FA = focused attention; '<0.1, p **p < 0.01, ***p < 0.001.
